# A Comparative Study of Hemodynamic Parameters Following Subarachnoid Block in Patients With and Without Hypertension

**DOI:** 10.7759/cureus.20948

**Published:** 2022-01-05

**Authors:** Amrita Panda, Manoja K Muni, Abhijeet Nanda

**Affiliations:** 1 Anesthesia, Kalinga Institute of Medical Sciences, Bhubaneswar, IND

**Keywords:** normotensives, bradycardia, hypertension, hypotension, subarachnoid block

## Abstract

Background

Hypotension is common in patients receiving a subarachnoid block (SAB) and can decrease vital organ perfusion, causing increased morbidity and mortality. Patients may receive intraoperative intravenous (IV) fluids and rescue medications to avoid these adverse effects and maintain adequate hemodynamic parameters. We conducted this study to compare the hemodynamic parameters and heart rate following spinal anesthesia on patients receiving antihypertensive medications (e.g., calcium channel blockers with or without beta-blockers) with those without hypertension during surgical procedures requiring SAB.

Methods

We conducted this two-year, single-center, prospective observational study in patients with hypertension and blood pressure within reference ranges receiving elective surgical procedures that do not require a definitive airway. All participants were from the American Society of Anesthesiologists physical status I and II. We assigned patients into three groups. Group 1 consisted of patients with hypertension receiving calcium channel blockers. Group 2 consisted of patients with hypertension receiving calcium channel blockers with beta-blockers. Patients whose blood pressure was within reference range were assigned to group 3. We collected patient ages and body weights, and we compared heart rate (HR), systolic and diastolic blood pressure (SBP; DBP), and mean arterial pressure (MAP) between the groups. We also compared the use of intraoperative rescue medications, IV fluid administration, and patient outcomes. We used Epi Info version 7.1 (Atlanta, GA: Centers for Disease Control and Prevention) public domain software for statistical analysis.

Results

Seventy-eight patients were included in the study (41 men, 37 women); group 1, HR, SBP, DBP, and MAP significantly declined over time in all groups, but there were no significant differences when comparing parameters between groups. Hypotension occurred in significantly more patients in group 1 (p=0.02) than groups 2 and 3. Bradycardia was experienced by significantly more patients in group 2 (p<0.05) than group 1 and group 3. Twenty-four patients in group 3, 17 patients in group 1, and 14 patients in group 2 required IV fluid in the first 20 minutes of the procedure, and the differences between the groups were not significant. Likewise, 15 patients in group 3, 14 in group 1, and 10 in group 2 required ephedrine, but the differences were not significant.

Conclusions

We compared intraoperative hemodynamic parameters following spinal anesthesia on patients receiving antihypertensive medications with patients without hypertension following SAB. Our results showed no meaningful differences in hemodynamic parameters between patients receiving antihypertensive drugs (calcium channel blockers without or in combination with beta-blockers) and those not receiving antihypertensive drugs after SAB. Therefore, calcium channel blockers alone or in combination with beta-blockers can be used to control patient blood pressure prior to surgery.

## Introduction

Subarachnoid block (SAB) is administered frequently in lower extremity surgery as a safe and effective alternative to general anesthesia. Hypotension is common following SAB, and the incidence increases with age. Antihypertensive medications in regular use can mitigate this effect. Intraoperative arterial hypotension has been associated with decreased perfusion to vital organs leading to increased mortality and morbidity. This effect can be enhanced due to impaired homeostasis in subjects with changes in the cardiovascular system's neurohumoral activity and a rapid increase in block height or simultaneous administration of drugs [1].

Labile blood pressure variations/fluctuations in hypertensive subjects and increased sympathetic activity during anesthesia are deleterious. The preservation of hemodynamic stability during anesthesia is a significant issue for anesthetists, especially for hypertensive patients. The risk of profound hypotension and associated management dilemma make anesthesiologists refuse to perform SAB in hypertensive subjects [2].

A paucity of data exists on the use of antihypertensive medications like calcium channel blockers alone or in combination with beta-blockers and their effects on hemodynamic parameters after SAB [3]. Therefore, this study aimed to compare hemodynamic parameters and assess the need for intravenous (IV) fluids and vasopressors following SAB between patients with hypertension and patients with blood pressure within the reference range.

## Materials and methods

This prospective observational study was conducted at the Department of Anesthesiology at Kalinga Institute of Medical Sciences, Bhubaneswar, Orissa, from September 2019 to September 2021. The study compared the intraoperative hemodynamic parameters after SAB in patients with hypertension treated with calcium channel blockers alone or with beta-blockers against patients with blood pressure within reference ranges receiving no antihypertensive medications. We also evaluated the use of IV fluids and rescue medications for hypotension following SAB within the first 30 minutes of the surgical procedure.

We calculated our sample size of 75 by considering power (1-beta) = 0.90 and alpha (type 1 error) = 0.05. The study included patients scheduled to receive elective surgical procedures lasting one to two hours without a definitive airway. Patients were eligible for inclusion in the study if they were aged 18 to 60 years, male or female, and were classified as American Society of Anesthesiologists (ASA) physical status I or II. Patients diagnosed with essential hypertension had to be receiving antihypertensive medications, including taking the medication on the day of the surgery. The study excluded patients with ASA III or IV on concomitant medications and those with associated comorbidities like diabetes, coronary artery disease or other cardiac diseases, severe hypovolemia, sepsis, and pregnancy. Patients were assigned into three groups. Group 1 consisted of patients with hypertension who received calcium channel blockers, group 2 consisted of patients with hypertension who received calcium channel blockers combined with beta-blockers, and group 3 patients had blood pressure within reference ranges and received no antihypertensive medications. All eligible patients were assessed for systolic blood pressure (SBP), diastolic blood pressure (DBP), mean arterial pressure (MAP), heart rate (HR), and average use of IV fluids, and the total dose of rescue medications to treat hypotension. A 30% decrease in MAP was defined as hypotension, and a 20% reduction in HR was considered bradycardia.

The study design followed the Declaration of Helsinki principles, received approval from the institutional ethics committee (#KIMS/KIIT/IEC/127/2019), and registered with the Clinical Trials Registry of India (#CTRI/2019/09/021473). All participants provided written informed consent.

Patient preparation

All enrolled subjects were preloaded 10 ml/kg of Ringer's lactate solution. On arrival to the preoperative holding area, patients received IV access with an 18G cannula. Standard ASA monitors were attached, and we recorded baseline values. We administered spinal anesthesia in aseptic conditions with the patient in a sitting position using a 25G Quincke needle (Franklin Lakes, NJ: Beckton Dickinson) in the L3-L4 space. Patients received 3 ml of 0.5% hyperbaric bupivacaine after the free flow of cerebrospinal fluid was established. The sensory level of T6 was achieved after SAB was performed. We then bolused patients with 10 ml/kg of IV fluids like crystalloids. 

Statistical analysis

We used Epi Info version 7.1 (Atlanta, GA: Centers for Disease Control and Prevention) public domain software for statistical analysis. Quantitative data were presented as arithmetic mean ± standard deviation. Qualitative data were presented as frequencies (%), and we considered p-values < 0.05 significant. We used the chi-square test to compare categorical variables. We use analysis of variance to assess intragroup variation and post hoc tests for variations over time.

## Results

Forty-one men and 37 women were included in the study for a total population of 78 patients. Patients in group 1 were significantly older (p=0.03) and heavier (p=0.03) than patients in group 2 and group 3. Table 1 presents the mean ages and body weights for all study groups.

**Table 1:** Demographic data SD: standard deviation; HR: heart rate; SBP: systolic blood pressure; DBP: diastolic blood pressure; MAP: mean arterial pressure

Parameters	Group 1 (n=25)	Group 2 (n=24)	Group 3 (n=29)	P-value
Age (years), mean ± SD	51.4 ± 12.3	48.5 ± 10.0	44.0 ± 7.8	0.03
Body weight (kg), mean ± SD	63.12 ± 10.78	56.58 ± 6.15	58.66 ± 8.79	0.03
Baseline HR (bpm), mean ± SD	79.40 ± 11.27	80.29 ± 8.49	77.66 ± 10.24	0.048
Baseline SBP (mm Hg), mean ± SD	130.72± 9.32	136.46 ± 9.63	127.59 ± 12.99	0.025
Baseline DBP (mm Hg) mean ± SD	83.20 ± 6.09	86.50 ± 6.05	81.72 ± 7.11	0.019
Baseline MAP (mm Hg) mean ± SD	83.64 ± 7.45	82.42 ± 6.17	74.03 ± 5.97	<0.001

**Figure 1:**
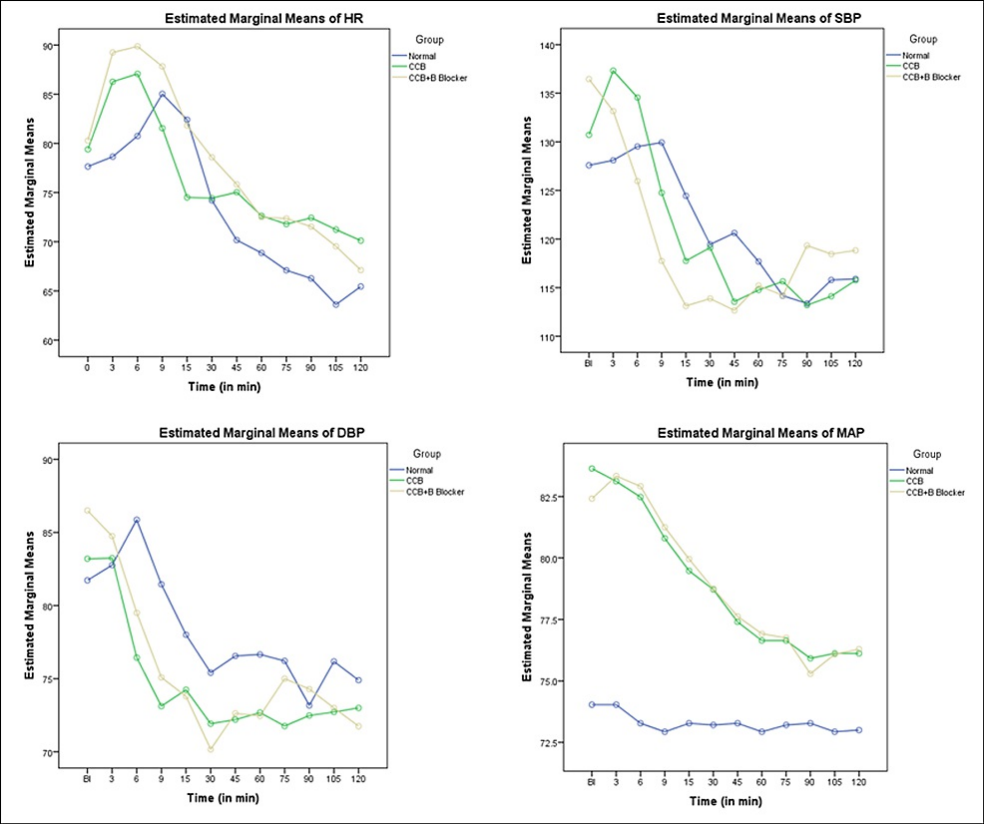
Estimated marginal means of different demographics (HR, SBP, DBP, and MAP) The graphs are showing (A) change in HR over time, (B) change in SBP over time, (C) change in DBP over time, (D) and change in MAP over time. SD: standard deviation; HR: heart rate; SBP: systolic blood pressure; DBP: diastolic blood pressure; MAP: mean arterial pressure; CCB: calcium channel blocker

Hemodynamic parameters including HR, SBP, DBP, and MAP were similar at baseline for all study groups as noted in Table 1. Hemodynamic parameters significantly decreased over time for all groups, but the differences between the groups were not statistically significant. Figure 1 presents the change in HR over time (Figure 1A), change in SBP over time (Figure 1B), change in DBP over time (Figure 1C), and change in MAP over time (Figure 1D).

The incidence of adverse effects is presented in Table 2. Significantly more patients in group 1 experienced hypotension following SAB (n=12) than patients in group 2 (n=3) and group 3 (n=3; p=0.02). However, significantly more patients in group 2 experienced bradycardia (n=10) than group 1 (n=0) and group 3 (n=3; p<0.05). Many patients in each group required ephedrine, and many required additional bolus of IV fluid in the first 20 minutes of the procedure, but the differences between the groups were not significant.

**Table 2:** Comparison of treatment received and adverse effects following SAB SAB: subarachnoid block

Parameters	Group 1 (n=25)	Group 2 (n=24)	Group 3 (n=29)	P-value
Hypotension incidence, n	10	2	3	0.02
IV fluid use in first 20 minutes, 1.5 L, n	17	14	24	0.09
Ephedrine use, n	14	10	15	0.47
Bradycardia, n	0	10	3	0.00

## Discussion

This study compared the hemodynamic parameters and HR following spinal anesthesia on patients receiving antihypertensive medications (e.g., calcium channel blockers with or without beta-blockers) with those of patients without hypertension during surgical procedures requiring SAB. We noted a positive relationship between age and hypertension in our findings, which aligned with reports in the literature [4]. We found no significant relationship between hypertension and gender, which aligned with findings from a previous study [5].

The hemodynamic effects of SAB also result in a sympathetic block of the venous reservoir [6]. This leads to venous pooling in the lowermost capacitance vessels [7]. When the sensory block extends greater T6 or farther, pooling in the hepatosplanchnic region can affect up to 20% of the circulating blood volume. Vasopressors counteract the hypotensive impact following SAB. Monotherapy with calcium channel blockers is effective in 50% of hypertensive patients, and combination therapy with beta-blockers is effective in 30-40% of hypertensive patients [8].

Patients with hypertension have an enhanced response to vasopressor drugs, and pulmonary edema can occur after fluid challenges [9]. Chronic untreated hypertension can injure the endothelium and cause vascular remodeling. Changes in arteriolar wall structure play a primary role in the patient's hemodynamic response to anesthesia, which explains the more remarkable changes in systemic vascular resistance and arterial pressure seen in patients with hypertension versus patients without hypertension with the same degree of the sympathetic blockade [10].

We saw a strong association between higher body weight and incidence of hypertension. There was no difference in baseline hemodynamic values among all three groups, which also aligned with the findings from a previous study [11]. There was no significant difference in HR changes over time across the three groups, but significance was observed in groups 1, 2, and 3 at nine minutes, 15 minutes, and 105 minutes, respectively. This decrease in HR could be due to the SAB effect reaching the T4 level and beyond combined with the effect of beta-blockers requiring atropine. Our results agreed with another study that reported that the incidence of bradycardia might increase with increasing age and level ≥T4 dermatomes [12].

In our study, the SBP decline over time was similar across all three groups. However, significant declines in SBP were observed in group 1, group 2, and group 3 at 15 minutes, 90 minutes, and 120 minutes after SAB, respectively. This could be due to inadequate preloading with fluids prior to SAB and the use of inadequate maintenance fluid or intraoperative loss. A previous study reported a significant difference regarding MAP and SBP between patients with controlled hypertension and patients with blood pressure within the reference range following SAB [13]. Similarly, the DBP showed no significant difference among the three groups at different time points in our study. Eight of 16 patients in group 2 required atropine. This could be due to the SAB reaching T4 or higher or the additive effect of the beta-blockers. Bradycardia was significant in group 2 due to the beta-blockers. The incidence of hypotension did not depend on the use of ephedrine.

A previous study used ultrasound-guided measurement of the inferior vena cava in 160 patients as an effective way to prevent post-spinal anesthesia hypotension [14]. Under SAB, patients with hypertension are more likely to develop hypotension than patients without hypertension.

Limitations

Our study had several key limitations. We could not evaluate the effect of antihypertensive medication in SAB based on the duration of treatment. However, we could evaluate the difference in hemodynamic parameters between monotherapy and combination therapy antihypertensive treatments. We could not corroborate body mass index with hypertension as we did not record patient height. We also did not use invasive arterial blood pressure monitoring or inferior vena cava ultrasonography to assess fluid responsiveness. We also did not record the number of times ephedrine was used.

## Conclusions

The study aimed to compare the variations of hemodynamics following SAB on patients with hypertension treated with calcium channel blockers alone or with beta-blockers against patients with blood pressure within reference ranges. Our results showed no meaningful differences in hemodynamic parameters between patients receiving antihypertensive drugs and those who did not receive antihypertensive drugs after SAB. Therefore, both monotherapy and combination therapy are equally effective in controlling blood pressure prior to surgery. However, patients using combination therapy are prone to bradycardia post-SAB. Early anticipation and treatment of hypotension in frail patients can be helpful for anesthetic management.

## References

[1] Olawin AM, Das JM (2021). Spinal anesthesia. StatPearls [Internet].

[2] Kavyashree NG, Danappa PM, Girish CN (2016). Comparison of haemodynamic profile after spinal anaesthesia in patients on regular treatment with calcium channel blockers and β blockers. J Evolution Med Dent Sci.

[3] Rothwell PM, Howard SC, Dolan E (2010). Effects of beta blockers and calcium-channel blockers on within-individual variability in blood pressure and risk of stroke. Lancet Neurol.

[4] Handschin A, Henny-Fullin K, Buess D, Leuppi J, Dieterle T (2015). Hypertension in the elderly. [Article in German]. Ther Umsch.

[5] Kaimar P, Sanji N, Upadya M, Mohammed KR (2012). A comparison of hypotension and bradycardia following spinal anesthesia in patients on calcium channel blockers and β-blockers. Indian J Pharmacol.

[6] Pitkänen M (2004). Density of spinal anaesthetic solutions. Br J Anaesth.

[7] Birnbach DJ, Browne IM (2010). Anesthesia for obstetrics. Miller's Anaesthesia. Seventh Edition.

[8] Tripathi KD (2013). Essentials of Medical Pharmacology. Seventh Edition. https://www.worldcat.org/title/essentials-of-medical-pharmacology/oclc/868299888?utm_medium=email&utm_source=transaction.

[9] Alemayehu TY, Berhe YW, Getnet H, Molallign M (2020). Hemodynamic changes after spinal anesthesia in preeclamptic patients undergoing cesarean section at a tertiary referral center in Ethiopia: a prospective cohort study. Patient Saf Surg.

[10] Martinez-Quinones P, McCarthy CG, Watts SW (2018). Hypertension induced morphological and physiological changes in cells of the arterial wall. Am J Hypertens.

[11] da Silva Neto WV, Azevedo GS, Coelho FO, Netto EM, Ladeia AM (2008). Evaluation of hemodynamic variations during anesthetic induction in treated hypertensive patients. Rev Bras Anestesiol.

[12] Kyokong O, Charuluxananan S, Sriprajittichai P, Poomseetong T, Naksin P (2006). The incidence and risk factors of hypotension and bradycardia associated with spinal anesthesia. J Med Assoc Thai.

[13] Gebrargs L, Gebremeskel B, Aberra B (2021). Comparison of hemodynamic response following spinal anesthesia between controlled hypertensive and normotensive patients undergoing surgery below the umbilicus: an observational prospective cohort study. Anesthesiol Res Pract.

[14] Ceruti S, Anselmi L, Minotti B, Franceschini D, Aguirre J, Borgeat A, Saporito A (2018). Prevention of arterial hypotension after spinal anaesthesia using vena cava ultrasound to guide fluid management. Br J Anaesth.

